# CHD1L: a novel oncogene

**DOI:** 10.1186/1476-4598-12-170

**Published:** 2013-12-21

**Authors:** Wen Cheng, Yun Su, Feng Xu

**Affiliations:** 1 Department of Urology, Nanjing Jinling Hospital, School of Medicine, Nanjing University, Nanjing 210002, Jiangsu, P.R. of China; 2 Outpatient department of Eastern Division, First Hospital of Nanjing, Nanjing Medical University, Nanjing, P.R. of China

**Keywords:** CHD1L, ALC1, Oncogene, Chr1q21, Amplification, ARHGEF9, SPOCK1, Nur77

## Abstract

Comprehensive sequencing efforts have revealed the genomic landscapes of common forms of human cancer and ~ 140 driver genes have been identified, but not all of them have been extensively investigated. *CHD1L* (chromodomain helicase/ATPase DNA binding protein 1-like gene) or ALC1 (amplified in liver cancer 1) is a newly identified oncogene located at Chr1q21 and it is amplified in many solid tumors. Functional studies of CHD1L in hepatocellular carcinoma and other tumors strongly suggested that its oncogenic role in tumorigenesis is through unleashed cell proliferation, G1/S transition and inhibition of apoptosis. The underlying mechanisms of CHD1L activation may disrupt the cell death program via binding the apoptotic protein Nur77 or through activation of the AKT pathway by up-regulation of CHD1L-mediated target genes (e.g., ARHGEF9, SPOCK1 or TCTP). CHD1L is now considered to be a novel independent biomarker for progression, prognosis and survival in several solid tumors. The accumulated knowledge about its functions will provide a focus to search for targeted treatment in specific subtypes of tumors.

## Introduction

Cancer is a disease of genome. International efforts in cancer genomic research have revealed that numerous somatic mutations, genomic rearrangement and structure variants in various type of cancer [1]. Approximately three to six genetic events are necessary to transform a normal cell into a cancer cell [2]. On average, two to eight somatic driver mutations occur in a typical tumor; the remaining are passenger mutations that confer no selective growth advantage. Critical genetic changes (in combinatory effect) reprogram the normal cell growth in several core signaling pathways to change the cell fate, cell survival and genome maintenance [1]. Driver genes are recently suggested to be categorized into “mut-driver genes” and “epi-driver genes”. Mut-driver genes contain a sufficient number or type of driver gene mutations; while epi-driver genes are expressed aberrantly in tumors but not frequently mutated; they are altered through changes in DNA methylation or chromatin modification that persist as the tumor cell divides [1]. A collection of all causalities of malignant transformation (also called the cancer initiatome [3]) measured with conventional molecular biological techniques or whole genome sequencing technologies will help us to find solutions to conquer cancer. Chromosomal rearrangements during tumorigenesis have been found to be common genomic abnormalities including amplifications, deletions or translocations that may result from a catastrophic shattering of one or more chromosomes followed by misjoining of the scrambled fragments upon repair, and kataegis [4].

Amplification of 1q21 is one of the most frequent genetic alterations in many solid tumors, including bladder [5], breast [6], nasopharyngeal carcinoma [7], hepatocellular carcinoma [8], esophageal tumor [9], fibrosarcoma of bone [10], colorectal carcinoma [11]. Chromodomain helicase/ATPase DNA binding protein 1-like gene (*CHD1L*) is a recently identified oncogene that is frequently amplified in hepatocellular carcinoma (HCC) [12]. CHD1L exhibits an oncogenic role during malignant transformation. Overexpression of CHD1L protein in tumors is considered to be a biomarker of poor prognosis and short tumor-free survival time. In this review, we will discuss more about the structure and function of CHD1L gene and its underlying molecular mechanisms during tumorigenesis. Finally, we will propose strategies for developing a CHD1L inhibitor for potential treatment.

### The structure of *CHD1L* gene

**Figure 1:**
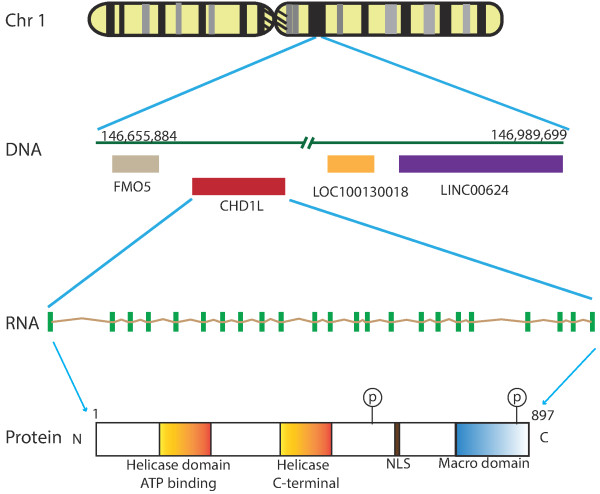
**Genomic information of human *CHD1L *gene (chromodomain helicase/ATPase DNA binding protein 1-like gene).** The genomic locus of *CHD1L* gene is located on the long arm q21 of Chromosome 1 with 53,152 base pairs in length. It is downstream of *FMO5* gene (flavin containing monooxygenase 5) and upstream of LOC10030018 (prostaglandin reductase 1 pseudo gene) and LINC00624. CHD1L contains 23 exons (green boxes), which may be transcribed with six transcript variants. The full-length transcript (NM_004284.2) and encoded protein structure is illustrated. CHD1L protein is comprised of two helicase domains (yellow color), a C-terminal macro domain (blue color) and nuclear localization sequence (NLS, purple color). There are two putative phosphorylation sites at the relative C-terminus of the protein: phospho-serine at 636 and 891 amino acids, respectively.

**Figure 2:**
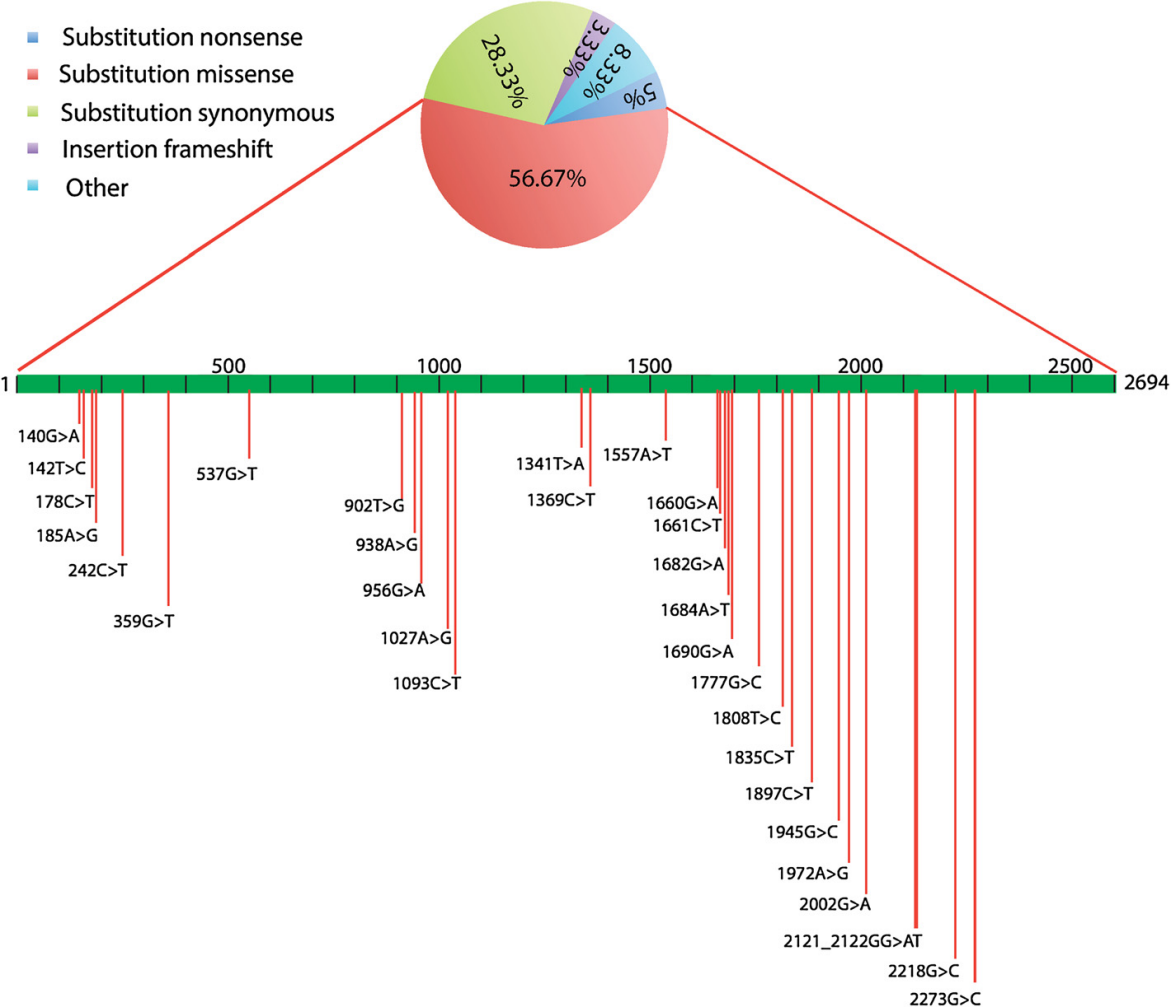
**The distribution of mutations in CHD1L.** The pie chart shows the percentage of mutations of substitution missense, nonsense, synonymous, insertion frame shift and others. The substitution missense mutations at the cDNA strand (2694 nucleotides) of CHD1L are displayed. The data is obtained from the catalogue of somatic mutations in cancer (COSMIC). (http://cancer.sanger.ac.uk/cosmic/gene/analysis?ln=CHD1L#dist).

Human *CHD1L* gene, also known as *ALC1* (amplified in liver cancer 1), was identified by Ma et al. in 2008 [12]. This gene is located at Chr 1q21.1 (genomic coordinate: chr 1:146,714,292-146,767,443, strand (+)). *CHD1L* gene is 53,152 base pairs long and it contains 23 exons. Upstream of *CHD1L* gene is flavin containing monooxygenase 5 (*FMO5*) gene, and downstream of *CHD1L* gene is prostaglandin reductase pseudogene (LOC100130018) and a long intergenic noncoding RNA 624 (LINC00624) (illustrated in Figure 1). Six alternatively spliced transcript variants have been described for this gene (http://www.ncbi.nlm.nih.gov/nuccore/?term=CHD1L). Interestingly, transcript variant 6 is a noncoding transcript; this is an example of coding RNAs that could serve as both coding and noncoding molecules depending on cellular context [13]. The corresponding proteins encoded from transcript variants are listed in Table 1. The full-length messenger RNA of *CHD1L* consists of 2,980 base pairs (3,036 bp in recent database) with a putative open reading frame coding an 897aa protein [12]. Protein sequencing analysis showed that CHD1L belongs to the SNF2-like family, containing a conserved SNF2_N domain, which is a helicase superfamily domain (helicase superfamily c-terminal domain (HELICc)), and a Macro domain [12] (Figure 1). The SNF2_N domain is composed of 280 amino acids, and the sequence homology between the SNF2_N domains of CHD1L and another SNF2-like family member, chromodomain helicase DNA binding protein 1 (CHD1), is 45% identical. The sequence homology of the HELICc domain (containing 107aa) between CHD1L and CHD1 is 59% identical [12]. Therefore, the name of chromodomain-helicase-DNA-binding protein 1-like, CHD1L, was given. A total of 64 different mutations have been reported in catalogue of somatic mutations in cancer (COSMIC) (http://cancer.sanger.ac.uk/cosmic/gene/analysis?ln=CHD1L#dist). These mutations are classified into substitution mutations (nonsense, missense, synonymous), insertion frameshift mutation and other (mutations occur at intronic regions). Among these mutations, the substitution missense mutations account for 56.67% as shown in the distribution chart. Since substitution missense mutations change the amino acids of the protein, and may affect the CHD1L functions. We listed these mutational locations at cDNA level (Figure 2). Recently, CHD1L mutations were detected in congenital anomalies of the kidneys and urinary tract (CAKUT) patient [14]. How these mutations change CHD1L biological functions in cancer cells remains to be explored. CHD1L expression was detected in different tissues using high density oligonucleotide microarray, in particular, it expresses at higher levels in early erythroid cells, CD34 cells, endothelial cells, dentritic cells and some leukemic cells (K562, HL60) [15]. Like other SNF2 chromatin remodeling proteins, CHD1L is localized to the nucleus.

**Table 1:** List of CHD1L transcript variants

**CHD1L mRNA**	**RefSeq #**	**Transcripts**	**Protein**	**notes**
Variant 1	NM_004284.4	3,036 bp	897 aa	Full length
Variant 2	NM_001256336.1	2,929 bp	803 aa	331-424 missing
Variant 3	NM_024568.2	3,008 bp	784 aa	1-113 missing
Variant 4	NM_001256337.1	2,506 bp	616 aa	44-246 missing
Variant 5	NM_001256338.1	2,424 bp	693 aa	43-246 missing
Variant 6	NR_046070.1	2,851 bp		Non coding transcript

### The functions of CHD1L

CHD1L is a recently identified oncogene located at 1q21, a frequently amplified region in hepatocellular carcinoma (HCC) [12]. The biochemical functions of CHD1L were predicted based on the protein structure similarity with CHD1. The CHD1 family of proteins is characterized by the presence of chromo (chromatin organization modifier) domains and SNF2-related helicase/ATPase domains. CHD1 protein is able to bind DNA and regulate ATP-dependent nucleosome assembly, modification of chromatin structure and mobilization through their conserved double chromodomains and SNF2 helicase/ATPase domain [16]. Sequence comparison showed that CHD1L contains SNF2-N domain and a helicase superfamily domain; therefore, CHD1L has also been hypothesized to play important roles in transcriptional regulation, maintenance of chromosome integrity and DNA repair. But unlike CHD1, CHD1L does not contain a chromodomain, which can recognize methylated histone tails. Instead, CHD1L contains a macro domain [12], which is an adenosine 5′-dephosphate (ADP)-ribose/polymer of ADP-ribose (PAR)-binding element [17]. Thus, CHD1L possesses a PAR-dependent chromatin remodeling activity and facilitates DNA repair reactions within a chromatin context [18]. ATPase and chromatin remodeling activities of CHD1L are strongly activated by the poly (ADP-ribose) polymerase Parp1 and its substrate NAD^+^ via transient interaction between intact macrodomain and chromatin-associated proteins, including histones and Parp1 [19]. This CHD1L nucleosome remodeling activity depends on the formation of a stable CHD1L-PARylated PARP1-nucleosome intermediate [20].

**Figure 3:**
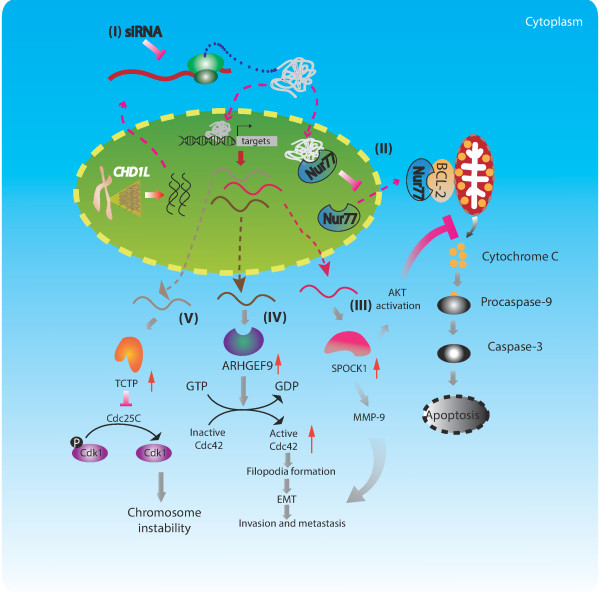
**The underlying mechanisms of oncogenic role of CHD1L during tumorigenesis.***CHD1L* is amplified at Chr1q21 region and overexpressed in tumors **(I)**. The macro domain of CHD1L protein interacts with Nur77 and inhibits the latter’s nuclear to mitochondrial translocation and the subsequent Nur77-mediated caspases’ activation and cell death **(II)**. On the other hand, CHD1L protein may directly bind to the promoter regions of target genes and activate gene transcription, such as ARHGEF9, TCTP and SPOCK1, leading to various biological effects such as cell survival, invasion, metastasis and genome instability **(III-V)**.

In addition to the PAR binding, the C-terminal macro domain (residues 600–897) of CHD1L is able to bind the protein Nur77: a critical member of a p53-independent apoptotic pathway. This binding subsequently inhibits the nucleus-to-mitochondria translocation of Nur77, which is the key step of Nur77-mediated apoptosis. Retention of Nur77 protein in the nucleus by CHD1L results in preventing release of cytochrome c from mitochondria and blocking the initiation of apoptosis [21] (Figure 3-(II)). Moreover, the chromatin-remodeling function of CHD1L plays important role in the earliest cell divisions of mammalian development [22].

CHD1L appears to function as a transcription factor. A chromatin immunoprecipitation-based cloning strategy unveiled that CHD1L confers DNA-binding capability to activate gene expression of direct targets that are relevant to oncogenesis. ARHGEF9 (Rho guanine nucleotide exchange factor 9), which encodes a specific guanine nucleotide exchange factor for the Rho small GTPase Cdc42, was identified as a CHD1L target gene [23]. CHD1L protein also directly binds to the promoter region (nt −733 to −1,027) of *TCTP* (translationally controlled tumor protein) [24] and the promoter region (nt −1662 to +34) of *SPOCK1* (sparc/osteonectin, cwcv, and kazal-like domains proteoglycan 1) [25], subsequently activating these target genes’ transcription. The transcriptional regulation of these genes by CHD1L could partially explain the mechanisms of CHD1L oncogenic role in cancer development which will be discussed in detail later. Collectively, CHD1L interacts with other proteins or regulates target gene expression to execute its biological effects.

### CHD1L and Cancer

Amplification of 1q21 region was reported in multiple solid tumors [5-7,9-11]. In hepatocellular carcinoma (HCC), amplification of 1q21 is the most frequent genetic alteration, being detected in 58%-78% of primary HCC cases by comparative genomic hybridization [8]. This phenotype leads cancer biologists to wonder why this region is amplified and what genes are misregulated in this region. In 2008, Ma et al. [12] first isolated *CHD1L* as a target gene within the 1q21 amplicon using chromosome microdissection/hybrid selection approach. Recently, several genes including CHD1L in regions for amplification at 1q21-24 in urothelial carcinoma were identified by array-CGH for high-resolution zoom-in oligonucleotide array analyses [26]. From HCC studies, CHD1L was not only detected to be amplified via FISH, but its mRNA and protein were also overexpressed in the examined samples [12]. Additionally, CHD1L-transfected cells possessed a strong oncogenic ability with increased colony formation in soft agar and the tumorigenity in nude mice. This phenotype could be effectively suppressed by small interfering RNA against CHD1L [12]. To further investigate the *in vivo* oncogenic role of CHD1L, a transgenic mouse model that ubiquitously expresses CHD1L was generated by Chen et al. [27]. Spontaneous tumor formation was found in 10/41 (24.4%) transgenic mice, including 4 HCCs, that were not found in their 39 wild-type littermates. Furthermore, overexpression of CHD1L in hepatocytes could promote tumor susceptibility in CHD1L-transgenic mice [27]. The oncogenic role of CHD1L in tumorigenesis *in vitro* and *in vivo* was also observed in colorectal carcinoma [11]. CHD1L expression in HPV-infected immortalized cervical cells appears to accelerate the malignant transformation with NNK chemical exposure [28]. All of this evidence strongly suggests that CHD1L functions as a driver gene during cancer development.

The clinical significance of amplification and overexpression of CHD1L have been evaluated in solid tumors, including HCC [29], ovarian carcinoma [30], colorectal carcinoma [11], and bladder cancer [31]. All these studies demonstrated that CHD1L is a novel biomarker for prediction of progression, prognosis and survival (Table 2). For example, we found that CHD1L protein expression was significantly higher in bladder cancer than in adjacent noncancerous tissues. CHD1L overexpression was significantly correlated with histologic grade and tumor stage. The Kaplan-Meier survival analysis revealed that survival time of patients with higher CHD1L expression was significantly shorter than that with lower CHD1L expression [31]. The role of CHD1L in chemotherapy response of patients with HCC was also investigated [32]. CHD1L could selectively inhibit apoptosis induced by 5-fluorouracil (5-FU) but not doxorubicin. The phenotype of chemo-resistance could be reversed by short hairpin siRNAs against CHD1L *in vitro* cell culture and *in vivo* mouse model [32]. Taken together, CHD1L is a novel oncogene and could be used as an indicator of poor prognosis and chemo-resistance.

**Table 2:** List of the studies of Chr1q and/or CHD1L amplification in cancer

**Tumor origin**	**Chr1q/CHD1L**	**Technique used**	**Biological/clinical impact**	**References**
Breast	Chr1q ( 67%)	CGH	genetic aberration	[6]
Bladder	Chr1q ( 54%)	CGH	Invasive cancer	[5]
	CHD1L	PCR	Poor prognosis	[31]
	Chr1q21-24	CGH + expression	Survival genes	[26]
Colorectal	CHD1L	FISH, PCR	Oncogene, Poor survival	[11]
Esophageal	Chr1q	CGH	Genetic aberration	[9]
Fibrosarcoma	CHD1L	CGH	Genetic defect	[10]
Liver	Chr1q21	CGH	Poor prognosis	[8]
	( 56 ~78%)	FISH	CHD1L was	[12]
	Chr1q21	TMA + IHC	Sequenced	[29]
	CHD1L		Poor prognosis	
	CHD1L			
Ovarian	CHD1L	TMA + IHC	Metastatic cancer with poor prognosis	[30]

### The mechanisms of CHD1L-driven oncogenesis

Driver genes (mut-driver genes or epi-driver genes) confer selective growth advantage that can be classified into 12 signaling pathways, which regulate three core cellular processes: cell fate, cell survival and genome maintenance [1]. Does the *CHD1L* gene have these features in tumor cells? Functional studies showed that overexpression of CHD1L could promote cell proliferation, accelerate G1/S phase transition and inhibit apoptosis [11,12]. In a transgenic mouse model, CHD1L could facilitate DNA synthesis and G1/S transition through the up-regulation of Cyclins (A, D1 and E), CDK2, 4, and down-regulation of Rb, p27 (Kip1), and p53 [27].

CHD1L-mediated transcriptional activation of target genes seems to play crucial roles during cancer development. Functional studies *in vitro* and *in vivo* showed that CHD1L contributed to tumor cell migration, invasion, and metastasis by increasing cell motility and inducing filopodia formation and epithelial-mesenchymal transition (EMT) via ARHGEF9-mediated Cdc42 activation. Therefore, CHD1L-ARHGEF9-Cdc42-EMT might be a novel pathway involved in HCC progression and metastasis [23] (Figure 3-(IV)).

As mentioned before, TCTP and SPOCK1 are direct transcriptional targets of CHD1L. CHD1L-mediated overexpression of TCTP was detected in 40.7% of human HCC samples. Clinically, overexpression of TCTP was significantly associated with the advanced tumor stage and overall short survival time of HCC patients. In multivariate analyses, TCTP was determined to be an independent marker associated with poor prognostic outcomes. Functional studies *in vitro* and *in vivo* demonstrated that TCTP has tumorigenic abilities, and overexpression of TCTP induced by CHD1L contributed to the mitotic defects in tumor cells. The mechanism of mitotic defect from overexpression of TCTP is that TCTP promotes the ubiquitin-proteasome degradation of Cdc25C during mitotic progression, which caused the failure in the dephosphorylation of Cdk1 on Tyr15 and decreased Cdk1 activity. As a consequence, the sudden drop of Cdk1 activity in mitosis induced a faster mitotic exit and chromosome missegregation, which led to chromosomal instability. Depletion of TCTP can prevent the mitotic defect. Collectively, CHD1L-TCTP-Cdc25C-Cdk1 is a novel molecular pathway, which causes the malignant transformation of hepatocytes with the phenotypes of accelerated mitotic progression and the production of aneuploidy [24] (Figure 3-(V)).

CHD1L-mediated upregulation of SPOCK1 can prevent apoptosis of HCC cells through activating Akt signaling pathway, blocking release of cytochrome c and activating caspase-9 and caspase-3. These effects were abolished with an Akt inhibitor. Additionally, HCC cells with overexpression of SPOCK1 have higher levels of matrix metallopeptidase 9, these cells were more invasive, and developed more metastatic nodules in immunodeficient mice than HCC cells with lower SPOCK expression [25] (Figure 3-(III)). Taken together, CHD1L activates cell survival pathways and inhibits programmed cell death signaling, resulting in cell fate change (malignant transformation) through complex mechanisms.

### Targeting CHD1L for potential treatment

Identification of cancer driver genes can lead to better diagnosis and successful targeted therapies. We are proposing here to develop small molecules to target degradation of oncogenic CHD1L gene and its encoded products. Hopefully this strategy could disrupt the putative pathways or counterparts, in turn, restore the normal cellular functions. Compelling experimental data *in vitro* and *in vivo* showed that knockdown of CHD1L gene expression using specific RNA interfering molecules could change the cancer cell behaviors through inducing apoptosis (Figure 3-(I)). Silencing CHD1L expression in HCC by the corresponding shRNA has a great therapeutic potential in HCC treatment, especially to increase the chemo-sensitivity combined with 5-FU chemotherapy [32]. siRNA-based therapy is emerging as a promising approach for a treatment. Several siRNA-mediated therapies are in clinical trial [33]. We propose that CHD1L-shRNA should be investigated for its utility as a targeted therapy based on the current preclinical evidence. Alternatively, because CHD1L contains a macrodomain, interacting with multiple counterparts (e.g., PARP1 and Nur77) to execute its biological effects; therefore, targeting macro domains might enhance the effectiveness of radiotherapy and chemotherapy [34]. The ideas could be: 1) utilizing PARP1 inhibitor; 2) designing small molecules to prevent Nur77 binding, resulting in increasing apoptotic pathways (Figure 3-(II)); 3) inhibiting target genes of CHD1L to inactivate downstream pathways (Figure 3-(III-V)). Ideally, combining all these strategies may have additive or synergistic effects. One might utilize the computer-aided drug design with high-throughput screening of known small molecule library (repositioning drug discovery) technology to achieve this goal in an efficient and inexpensive manner.

## Conclusion

Since CHD1L gene was isolated from the amplicon of Chr1q21 in tumors, the functional studies point to the oncogenic role of CHD1L in solid tumors, particularly in hepatocellular carcinoma. The unique protein structure of CHD1L with macro domain interacting with other protein partners executes a variety of biological functions such as DNA damage repair and anti-apoptosis. Moreover, CHD1L-mediated gene activation may confer regulatory function in malignant transformation. Better understanding of CHD1L genomic functions will likely pave the way for novel therapeutic strategies (siRNA, small molecules) to modulate critical signaling pathways in cancer.

## Abbreviations

ALC1: Amplified in liver cancer 1; ARHGEF9: Rho guanine nucleotide exchange factor 9; CHD1L: Chromodomain helicase/ATPase DNA binding protein 1-like gene; CGH: Comparative genomic hybridization; FISH: Flurosecent in Situ hybridization; HCC: Hepatocellular carcinoma; IHC: Immuno histo chemistry; SPOCK1: Sparc/osteonectin, cwcv, and kazal-like domains proteoglycan; TMA: Tissue microarray; TCTP: Translationally controlled tumor protein.

## Competing interests

The authors declare that they have no competing interests.

## Authors’ contributions

WC, YS and FX conceived this review, YS and FX drafted the manuscript; WC supervised and gave final approval of this version to be published. All authors read and approved the final manuscript.
